# Correlations Between the Neutrophil-Lymphocyte Ratio, Platelet-Lymphocyte Ratio, and Serum Lipid Fractions With Neovascular Age-Related Macular Degeneration

**DOI:** 10.7759/cureus.62503

**Published:** 2024-06-16

**Authors:** Diana F Tricorache, Ana M Dascalu, Cristina Alexandrescu, Anca Bobirca, Catalin Grigorescu, Corneliu Tudor, Bogdan M Cristea

**Affiliations:** 1 Medicine, Carol Davila University of Medicine and Pharmacy, Bucharest, ROU; 2 General Surgery, Grinvest Concept Company, Bucharest, ROU

**Keywords:** neutrophil-to-lymphocyte ratio (nlr), platelet-to-lymphocyte ratio (plr), anti-vegf therapy, central macular thickness (cmt), hdl-cholesterol, lipid metabolism, neovascular age-related macular degeneration

## Abstract

Introduction

Age-related macular degeneration, a chronic and progressive disease, is one of the leading causes of vision loss globally among the elderly population. Multiple hypotheses have been proposed regarding its pathogenesis, including the presence of lipid metabolism alteration. Dysfunctional lipid handling within retinal pigment epithelial cells has been implicated in the accumulation of lipofuscin and subsequent induction of oxidative stress and inflammation, all contributing to retinal degeneration. The present study aims to comparatively analyze the serum lipid fraction distributions in patients with neovascular age-related macular degeneration (AMD) and controls.

Materials and methods

A retrospective study was carried out between January 2021 and December 2023 on 91 naïve patients with neovascular AMD and 90 controls admitted for routine cataract surgery. All subjects underwent a comprehensive ophthalmological exam, including ophthalmoscopy and optical coherence tomography (OCT) with central macular thickness (CMT) measurement. A complete blood count with differential and lipid fractions values was analyzed. The neutrophil-lymphocyte ratio (NLR), platelet-lymphocyte ratio (PLR), total cholesterol (TC), high-density lipoprotein cholesterol (HDL-C), low-density lipoprotein cholesterol (LDL-C), and triglycerides (TG) were comparatively analyzed between the control group and the test group.

Results

The groups were comparable in terms of age (73.84 ±7.52 years for the neovascular AMD group vs 72.1±10.92 years in controls; p=0.8) and gender distribution (p=0.243). The mean NLR and PLR values were slightly higher in the AMD group but not statistically significant (p=0.51, p>0.99, respectively). Comparative analysis of lipid profile fractions showed significantly higher HDL-C values in the exudative AMD group compared to normal subjects (61.27±19.4 mg/dL vs 50.99±7.86 mg/dL, p=0.006). Also, the proportion of subjects with HDL-C>60 mg/dL was higher in the exudative AMD group (p=0.014). There were no significant differences in total cholesterol (189.77±53.39 mg/dL vs 190.43±37.84 mg/dL, p=0.681), LDL-C, and TG. Logistic regression analysis showed that serum HDL-C and HDL-C values >60 mg/dL are significantly associated factors with neovascular AMD. However, there is no statistical correlation between the values of these biochemical parameters and visual acuity or CMT in the neovascular AMD patient group.

Conclusions

There were no correlations between NLR and PLR with neovascular AMD in the study group. Higher HDL-C values exceeding 60 mg/dL were associated with neovascular age-related macular degeneration and could represent a possible therapeutic target in neovascular AMD.

## Introduction

Age-related macular degeneration is a multifactorial eye disease involving complex mechanisms such as hypoxia, inflammation, vascular insufficiency, oxidative stress, and lipid dyshomeostasis, all leading to progressive degeneration of photoreceptors, retinal pigment epithelium (RPE), and Bruch's membrane [1]. Early AMD is characterized by the presence of drusen, which are accumulations of lipids at the level of an inner collagenous layer of Bruch's membrane [2]. The presence of multiple drusen is a risk factor for disease progression to either the dry/nonexudative form or the exudative form, both having disastrous effects on vision loss. Altered homeostasis of lipid metabolism has recently emerged as a potential physiopathological mechanism, being supported by numerous studies, including biochemical studies, genetic studies, and studies revealing altered plasma lipid levels in AMD patients [3-7]. This theory is supported by the abundant presence of lipids in drusen, polymorphisms of the apolipoprotein-E (APO-E) gene, and the involvement of dyslipidemia as a well-known risk factor in AMD.

Multiple histopathological studies on both human and mouse retinas helped highlight the role of cholesterol at the retinal level [3,5,7-9]. These studies have not only demonstrated the presence of cholesterol in drusen, basal linear deposits, basal laminar deposits, and subretinal drusenoid deposits - all features of age-related macular degeneration - but also the presence of intrinsic cholesterol synthesis by the neural retina and retinal pigment epithelium (RPE) [3,7-9]. Interestingly, cholesterol from systemic circulation can cross RPE and reach the neural retina, the transport involving lipoproteins such as high-density lipoprotein (HDL) and low-density lipoproteins (LDL) [5,8]. Specific HDL and LDL receptors exist in the basal labyrinth of RPE through which HDL and LDL enter and deliver lipophilic nutrients such as vitamin A, E, lutein, and unesterified cholesterol, all of these being involved in the assembly of apolipoprotein B (ApoB) and apolipoprotein E (ApoE) lipoproteins [5,8,10]. With the help of microsomal triglyceride transfer protein (MTP), which RPE expresses, secretion of esterified cholesterol particles from ApoB occurs in Bruch's membrane (BrM), where they are retained and eventually cleared by diffusion through choriocapillaris endothelium [11-15]. With aging, choriocapillaris endothelium is unable to maintain clearance, and lipoproteins begin to accumulate, creating a lipid wall on the BrM inner surface known as pre-basal linear deposits (preBLinD), which are the precursors for AMD-specific lesions [5,8,9,16].

Conflicting results emerged from studies aimed at finding causal relationships between lipid biomarkers and AMD [17-22]. Chakravarthy et al. [23], in a meta-analysis of 24 studies including 113,780 persons with 17,236 cases of late AMD, found no clear correlation between serum lipid fractions and the risk of AMD. A UK prospective cohort study and a Mendelian randomization study both identified HDL as a causal risk factor for AMD without finding similar associations for LDL or triglycerides (TG) [24]. Nonetheless, some recent cross-sectional and cohort studies have reported no significant associations between serum lipoprotein profiles and AMD [25, 26]. A systematic review by Wang et al. [6] identified a significantly increased risk of AMD associated with higher serum HDL-C levels, with a relative risk (RR) of 1.18 per 1 mmol/L increase, while elevated serum total cholesterol (TC), LDL-C, and TG were linked to a decreased risk of AMD. These findings challenge the conventional perspective that HDL is "good" cholesterol and LDL is "bad" cholesterol, as typically understood in the context of cardiovascular disease [27]. One explanation may be that in higher concentrations, HLD-C may slow the process of cleaning and may also be exposed to oxidation in the highly oxidative environment of the macula. Reactive oxygen species (ROS) from neighboring mitochondria create a pro-inflammatory and toxic environment, where lipoproteins will fuse and form lipid pools, rendering them unstable and cytotoxic eventually leading to activation of the inflammatory cascade [13-16].

In the present analysis, we aim to comparatively analyze the systemic inflammatory biomarkers NLR and PLR, as well as the serum lipid fractions in patients with neovascular AMD versus healthy controls. Furthermore, we searched for correlations between these paraclinical parameters and the visual acuity and central macular thickness in the neovascular AMD group.

## Materials and methods

Subjects and inclusion criteria

A retrospective study was carried out between January 2021 and December 2023, including patients admitted with neovascular AMD in the Department of Ophthalmology, Emergency University Hospital, Bucharest, undergoing anti-vascular endothelial growth factor therapy (anti-VEGF) therapy. The study was conducted in agreement with the Declaration of Helsinki, and it was approved by the Ethical Committee of The University Emergency Hospital Bucharest (No. 13966/01.03.2024). All subjects gave informed written consent after a reasonable disclosure [28]. During the COVID-19 pandemic, the regulations regarding frequent disinfection and decontamination, social distancing, and wearing masks were strictly obeyed [29]. The diagnosis of neovascular AMD was established after a complete ophthalmologic exam and documentation of macula thickness and morphology by optical coherence tomography (Cirrus 5000 HD OCT, Macular Cube program; Carl Zeiss AG, Baden-Württemberg, Germany). The inclusion criteria for the neovascular AMD group were the presence of subretinal fluid, cystic maculopathy, or central macular thickness (CMT) of at least 250 µm, as detected by OCT with no prior anti-VEGF therapy. The control group consisted of patients admitted for routine cataract surgery with normal macular morphology.

Subjects with pre-existing systemic conditions (e.g., treated dyslipidemia, diabetes, lymphoproliferative diseases, connective tissue diseases, chronic hepatic or renal failure, malignancies) and ocular pathologies (glaucoma, other causes of subretinal neovascularization, macular edema, impairment of fundus examination by anterior segment opacities) were excluded from this study.

For all subjects, best corrected visual acuity (BCVA), CMT, and paraclinical data, including a complete blood count with differential and lipid fractions values, were extracted from medical sheets and electronic patient records.

Neutrophil-lymphocyte ratio (NLR), platelet-lymphocyte ratio (PLR), total cholesterol (TC), high-density lipoprotein cholesterol (HDL-C), low-density lipoprotein cholesterol (LDL-C), and triglycerides (TG) were analyzed in terms of means±SD and compared with the control group. For neovascular AMD subjects, BCVA and CMT were cross-referenced with serum lipids to check for correlation.

Statistical analysis

Numeric variables were expressed as mean (±SD) and discrete outcomes as absolute and relative (%) frequencies. We created two groups according to the presence of nAMD. Group comparability was assessed by comparing baseline demographic data and follow-up duration between groups. Normality and heteroskedasticity of continuous data were assessed using Shapiro-Wilk and Levene's test, respectively. Continuous outcomes were compared with unpaired Student t-test, Welch t-test, or Mann-Whitney U test according to data distribution. Discrete outcomes were compared with chi-squared or Fisher's exact test accordingly. The alpha risk was set to 5%, and two-tailed tests were used. A logistic regression was performed to assess the relation between neovascular AMD and the explanatory variables NLR, PLR, total cholesterol, TG, LDL-C, VLDL-C, and HDL-C. Data were checked for multicollinearity with the Belsley-Kuh-Welsch technique. Heteroskedasticity and normality of residuals were assessed respectively by the Breusch-Pagan test and the Shapiro-Wilk test. A p-value of 0.05 was considered statistically significant. Statistical analysis was performed with EasyMedStat (version 3.30.2). ANOVA statistical analysis was performed to assess the correlations between the studied biological parameters, BCVA, and CMT in the neovascular AMD group.

## Results

A total of 181 subjects were included in this study, divided into a control group (90 subjects, 49.8%) and a neovascular AMD group (91 subjects, 50.2%). There were no differences between the neovascular AMD group and controls in terms of age (72.1 ±10.92 years vs 73.84 ±7.52 years, p=0.8) and sex distribution (p=0.24; Table 1).

**Table 1:** Demographic and paraclinical data of patients included in neovascular AMD vs control group ^a^ Unpaired Student t-test; ^b^ p-value was calculated by chi-squared test; ^c^ Mann-Whitney test; Alpha risk was set to 5% (α = 0.05); ^d^ Fischer exact test; * p value <0.05. This value was considered statistically significant for all tests. AMD - age-related macular degeneration; BSVA - best corrected visual acuity; CMT - central macular thickness; PLR - platelet-lymphocyte ratio; NLR -  neutrophil-lymphocyte ratio; HDL-C - high-density lipoprotein cholesterol; LDL-C - low-density lipoprotein cholesterol; VLDL - very-low-density lipoprotein; TG - triglycerides

Variable	Neovascular AMD, N=91	Controls, N=90	p-value
Age in years (mean ± SD)	73.8 ± 7.5	72.1 ± 10.9	0.8^a^
Male sex, N (%)	53 (58.2%)	56 (62.2%)	0.76^b^
BCVA (mean ± SD)	0.3 ± 0.2	0.4 ± 0.5	0.08^c^
CMT (µm, mean ± SD)	393.4 ± 139.5	247.5 ± 23.3	<0.001^c^
Leukocytes *cells× 10^9^/L, mean ± SD)	7.5 ± 1.7	7.3 ± 1.7	0.69^a^
Lymphocytes (cells× 109/L, mean ± SD)	2.0 ± 0.7	1.9 ± 0.6	0.57^c^
Neutrophils (cells× 109/L, mean ± SD)	4.7 ± 1.4	4.5 ± 1.3	0.72^c^
Thrombocytes (cells× 109/L, mean ± SD)	252.1 ± 55.7	239.4 ± 60.3	0.39^a^
PLR (mean ± SD)	142.8 ± 75.3	132.3 ± 43.1	>0.99^c^
NLR (mean ± SD)	2.7 ± 1.7	2.5 ± 0.9	0.51^c^
Total cholesterol (mg/dL, mean ± SD)	189.7 ± 53.4	190.4 ± 37.8	0.68^c^
HDL-C (mg/dL, mean ± SD)	61.2 ± 19.4	50.9 ± 7.8	0.006^c^*
HDL-C>60 (mg/dL, mean ± SD)	19 (61.29%)	8 (26.67%)	0.01^d^*
LDL-C (mg/dL, mean ± SD)	101.6 ± 35.4	115.3 ± 39.1	0.15^a^
VLDL (mg/dL, mean ± SD)	26.6 ± 17.4	24.1 ± 8.8	0.88^b^
LDL-C/HDL-C ratio (mean ± SD)	1.7 ± 0.7	2.3 ± 0.9	0.003^b^*
TG (mg/dL, mean ± SD)	136.7 ± 88.5	120.5 ± 44.0	0.98^b^

**Figure 1:**
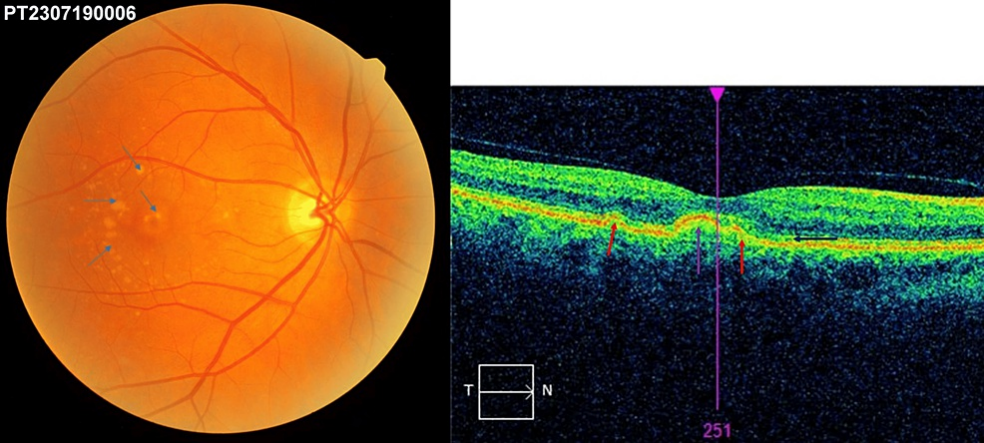
Right) fundus photography - multiple confluent soft drusen at the macular region (blue arrows); left) optical coherence tomography macular scan showing sub-retinal pigment epithelium (RPE) drusen deposits with medium, homogeneous internal reflectivity (red arrows); drusenoid pigment epithelium detachment (purple arrow); and subretinal drusenoid deposits - SDD (black arrow)

The mean CMT measured by optical coherence tomography (OCT) macular cube analysis was significantly higher in the neovascular AMD group compared to controls (393.4±139.5 µm vs 254.5 ± 23.3 µm, p<001; Figure 1).

There were no statistically significant differences in terms of total leukocytes, lymphocytes, neutrophils, thrombocytes, NLR, and PLR between the neovascular AMD group and controls (Table 1). The results suggest that, while inflammation is a recognized factor involved in AMD pathogenesis, the changes could be moreover local, with a limited systemic involvement.

Comparative analysis of lipid profile fractions showed significantly higher HDL-C values in the neovascular AMD group compared to control (61.27±19.4 mg/dL vs 50.99±7.86 mg/dL, p=0.006). Also, the proportion of subjects with HDL-C>60 mg/dL was higher in the AMD group (p=0.01), while the LDL-C/HDL-C ratio was significantly lower (p=0.003).

A logistic regression analysis was performed to assess the relation between neovascular AMD and the explanatory variables HDL-C and HDL-C>60mg/dL. Data were checked for multicollinearity with the Belsley-Kuh-Welsch technique. A p-value of 0.05 was considered statistically significant (Table 2, 3).

**Table 2:** Logistic regression analysis between neovascular AMD and HDL-C *p-value <0.05 was considered statistically significant AMD - age-related macular degeneration; HDL-C - high-density lipoprotein cholesterol

Variable	Odds ratio	p-value
Intercept	0.00929 (0.000252;0.342)	0.011*
HDL-C (mg/dL)	1.09 (1.02;1.16)	0.0107

**Table 3:** Logistic regression analysis between neovascular AMD and HDL-C>60mg/dL * p-value <0.05 was considered statistically significant AMD - age-related macular degeneration; HDL-C - high-density lipoprotein cholesterol

Variable	Odds ratio	p-value
Intercept	0.545 (0.27;1.1)	0.0912*
HDL-C>60 mg/dL	4.35 (1.47;12.89)	0.00787

In regression analysis, HDL-C was associated with higher rates of neovascular AMD for each 1mg/dL increase (OR=1.09, p= 0.0107).

Moreover, the regression analysis found that values of HDL-C>60mg/dL were associated with higher rates of neovascular AMD (OR=4.35, p= 0.0079).

Furthermore, we analyzed if serum lipid fractions could be predictors for functional or structural changes in patients with neovascular AMD included in the study, using ANOVA analysis of variance. However, no correlation was found between HDL-C levels and HDL>60 mg/dL and BCVA (p=0.38 and p=0.34, respectively) or CMT (p=0.370 and p=0.615, respectively) in neovascular AMD patients. These data suggest that while higher values of HDL-C could be associated with an increased risk of neovascular AMD, it cannot be used as a biomarker of disease severity.

## Discussion

Age-related macular degeneration is one of the leading causes of irreversible vision loss in adults, which is only expected to rise with the aging population [30]. While anti-VEGF therapy is a relatively safe and efficient procedure that can be performed on a daycare basis, the control of the disease requires numerous hospital visits, which puts a serious burden on the patients and their families [31, 32]. Current research focuses on identifying alternative therapeutic targets, which may help in reaching a better control of the disease and spacing the interval between the intravitreal injections.

Several studies postulate that in conditions associated with AMD pathobiology, including advanced age, dietary factors, smoking, complement activation, CFH variants, and alterations in RPE metabolic function, there is an elevation in both the concentration and content of sub-retinal pigment epithelium (sub-RPE) HDL-like lipoproteins [16,33,34]. Moreover, alterations in HDL protein composition led to a transition from an anti-inflammatory and antioxidant state to an inflammatory one, potentially resulting in local sequestration. This transition may be attributed to modifications in proteins and lipids, leading to heightened oxidation, inflammatory responses, and diminished efficiency in removing cholesterol and lipids from cells. These pathophysiological alterations ultimately inflict damage on the RPE and its surrounding environment, likely contributing to the development and progression of AMD [34].

Genetic evidence of lipid metabolism involvement in AMD is supported by polymorphisms in multiple genes such as apolipoprotein E (ApoE), hepatic lipase (LIPC), cholesterol ester transfer protein (CETP), lipoprotein lipase (LPL) and ATP binding cassette subfamily A member 1 (ABCA1), all being identified as risk factors for development of AMD [17-19,22,35]. Two genome-wide association studies (GWAS) have linked AMD to variants in the hepatic lipase (LIPC), cholesterol ester transfer protein (CETP), ATP-binding cassette subfamily A member 1 (ABCA1), and lipoprotein lipase (LPL) genes [36-38]. A more recent and extensive GWAS involving 16,144 patients and 17,832 controls confirmed these associations, identifying variants in the ABCA1, ApoE, CETP, and LIPC genes [39]. Additionally, a meta-analysis of multiple GWAS reaffirmed that many loci associated with AMD are located near or within genes involved in high-density lipoprotein particle remodeling, cholesterol transporter activity, and macrophage foam cell differentiation, among other functions [27, 40].

Previous studies on serum lipoprotein profiles in AMD yielded inconsistent results, but recently, a tendency to report an association between high HDL and increased risk of AMD has emerged [20,21]. Supporting these observations, a multi-center European study of 32,483 subjects found that higher serum HDL-C was associated with an increased risk of AMD (OR: 1.21 per 1 mmol/L), while higher TG levels were associated with a decreased risk of AMD (OR: 0.94 per 1 mmol/L; 95% CI: 0.91-0.97) [41]. Similarly, the Tsuruoka Metabolomics Cohort Study in Japan reported that in male participants, increased HDL was significantly associated with higher AMD risk (OR: 1.61 per 1 mmol/L; 95% CI: 1.17-2.23) and decreased TG with lower AMD risk (OR: 0.78 per 1 mmol/L; 95% CI: 0.64-0.96) [42]. Our study confirms this finding, suggesting a correlation between neovascular AMD and HDL levels, with the latter being a potential risk factor. Regarding total cholesterol, LDL-C, and triglycerides, the present study found no correlation. 

The primary function of the HDL-C particles is to facilitate the efflux of cholesterol and phospholipids from cells and transport them to the liver for elimination. Additionally, HDL particles exhibit anti-inflammatory [34,43,44] and antioxidant properties [45, 46]. However, it has been observed that under certain conditions, such as cardiovascular disease [47], aging [48], or during an acute phase response, HDL can shift from an anti-inflammatory to a pro-inflammatory molecule [49]. This shift is primarily attributed to dynamic changes in the HDL proteome in these conditions. It remains unclear whether these alterations in the plasma HDL proteome contribute to the disease or are a result of it. It is possible that local concentrations and the specific composition of HDL particles in the eye, rather than circulating levels of HDL-cholesterol, may contribute to a damaging phenotype in AMD.

The occurrence of deposited HDL in the posterior segment of the eye appears to correlate with age, although the precise factors initiating this process remain unidentified. It is plausible that age-related changes in Bruch's membrane (BrM), resulting in decreased permeability to plasma proteins, lipids, and oxygen, may impede the transit of locally synthesized lipoproteins into circulation. Additionally, studies have demonstrated alterations in BrM glycosaminoglycans (GAGs) with aging [50], potentially facilitating lipoprotein binding in older individuals. It is evident that the accumulation of lipid- and protein-rich material in various ocular deposits, such as soft drusen, basal linear deposits, and subretinal drusenoid deposits, detrimentally impacts the health of retinal pigment epithelium (RPE) and photoreceptors. Decreasing the burden of these deposits could improve ocular health and potentially delay the progression of advanced AMD. The findings presented in this study lend support to this proposition and provide a proof of concept for the therapeutic targeting of HDL in AMD management [34].

Although a consensus regarding the exact threshold over which HDL-C becomes harmful has not been reached, our findings reveal that levels above 60 mg/dL are associated with wet AMD, a level above which we found most of our patients with advanced disease.

The current cardiovascular guidelines, where high levels of HDL-C exceeding 60 mg/dL are encouraged, need to be taken into consideration when addressing the special subcategory of AMD patients [51]. 

Furthermore, recent findings showed that higher levels of HDL-C may be associated with moreover with adverse acute cardiac events by slowing the process of cleaning LDL-C. Recent studies found that values increased over 60 mg/dL of HDL-C were related to a 50% increase in the chances of dying from heart attack [52-54].

Changing the culture in healthcare is a longstanding process and could be challenging [55]. If enough evidence can be gathered to support the causal relationship between HDL-C and advanced AMD, then there could be a clear benefit in revisiting said guidelines and including a special category for AMD patients, where both cardiovascular benefits and ophthalmological risks are taken into account, and a new target level is established.

Although HDL-C has been considered "good cholesterol" for many years, recent studies tend to disagree with this dogma [56, 57]. High HDL-C levels have been associated with an increased risk of dementia and infection [56,58,59]. Moreover, research in the cardiovascular field converges to the analysis of the particular types of protein subspecies of HDL-C [60]. Subspecies containing proteins related to inflammation, immune response, thrombosis, and lipid metabolism seem to correlate with an increased risk of cardiovascular disease, while those containing apolipoprotein C1 or E are linked with a reduced risk [57-60]. Therefore, assessing cardiovascular risk shouldn't rely solely on overall HDL levels but also consider the equilibrium between advantageous and disadvantageous HDL subspecies and their specific distribution. 

Causes for increased HDL-C may be genetic, implying cholesteryl ester transfer protein (CETP) deficiency and, a diet rich in alcohol and saturated fats, which may increase both LDL and HDL cholesterol. Medications such as statins, hormone replacement therapy for menopause, antiepileptic drugs, and thyroid hormones may lead to lipidic metabolism imbalances, with rise in HDL-C levels [52-54]. Statins play a crucial role as a lipid-lowering medication, but they also have a documented effect on boosting HDL-C levels, which would normally be a beneficial outcome [57]. However, in the context of a neovascular AMD patient, this outcome could ultimately prove harmful. With several studies already proposing that statins present a dose-dependent risk on AMD progression, and others a dose-dependent regression with the higher therapeutic doses, one must consider the scenario in which all patients receiving such lipid-lowering agents might benefit from AMD screening [57, 61-66]. Further research is necessary to establish the exact role of statins in AMD progression. Reducing alcohol and promoting a low-fat diet, as well as changing statin medication with another lipid-lowering agent, could be useful in a personalized approach to the patient with AMD exhibiting higher levels of HDL-C [52-54].

There are only a few studies on the correlations between NLR, PLR, and neovascular AMD. The researchers found higher NLR and PLR among neovascular AMD patients, directly correlated with CMT and inversely correlated with BCVA and response to anti-VEGF therapy [67-70]. Furthermore, Vergroesen et al. [70] found a correlation between NLR levels and the consumption of a pro-inflammatory diet in the neovascular AMD group. In our study, we found slightly higher values of NLR and PLR inflammatory biomarkers in the neovascular AMD group when compared to healthy controls; however, they were not statistically significant.

The present study has some limitations. It was a retrospective monocentric study on a relatively limited number of subjects. All patients included were Caucasians. The studied biomarkers were assessed at the initial admission; thus, a potential correlation with the response to anti-VEGF therapy was not evaluated in the present paper.

## Conclusions

There were no correlations between NLR and PLR with neovascular AMD in the study group. Serum HDL-C was higher in the neovascular AMD patients compared to healthy individuals in the same age group. Further studies are necessary to establish a concrete causality between AMD progression and certain levels of HDL-C so that some benefits could arise from maintaining a level below that threshold. Therefore, HDL-C may also represent a therapeutic target for long-term AMD management. Although further investigation is warranted, therapies aimed at restoring lipid balance to tackle the underlying cellular and molecular abnormalities of AMD would represent a significant breakthrough for countless AMD patients.
